# Prevalence and Antimicrobial Resistance of *Paeniclostridium sordellii* in Hospital Settings

**DOI:** 10.3390/antibiotics11010038

**Published:** 2021-12-29

**Authors:** Hanane Zerrouki, Sid-Ahmed Rebiahi, Yamina Elhabiri, Ahlam Fatmi, Sophie Alexandra Baron, Isabelle Pagnier, Seydina M. Diene, Jean-Marc Rolain

**Affiliations:** 1 Laboratoire de Microbiologie Appliquée à l’Agroalimentaire, au Biomédical et à l’Environnement, Université de Tlemcen, Tlemcen 13000, Algeria; zerrouki_hanane@hotmail.com (H.Z.); sido8472@yahoo.fr (S.-A.R.); yambio19@gmail.com (Y.E.); 2 Faculté de Médecine et de Pharmacie, 19-21 Boulevard Jean Moulin, CEDEX 05, 13385 Marseille, France; sophie.baron@ap-hm.fr (S.A.B.); isabelle.pagnier@univ-amu.fr (I.P.); 3 IRD, APHM, MEPHI, IHU-Méditerranée Infection, Aix Marseille Université, 13005 Marseille, France; 4 Laboratory of Applied Molecular Biology and Immunology, University of Tlemcen, W0414100, Tlemcen 13000, Algeria; ahlem.fatmi@gmail.com

**Keywords:** *Paeniclostridium sordellii*, hospital environment, MICs, antibiotics, disinfectant products, antimicrobial resistance

## Abstract

(1) Background: The purpose of this study was to determine the prevalence of clostridia strains in a hospital environment in Algeria and to evaluate their antimicrobial susceptibility to antibiotics and biocides. (2) Methods: Five hundred surface samples were collected from surfaces in the intensive care unit and surgical wards in the University Hospital of Tlemcen, Algeria. Bacterial identification was carried out using MALDI-TOF-MS, and then the minimum inhibitory concentrations (MICs) of various antimicrobial agents were determined by the E-test method. *P. sordellii* toxins were searched by enzymatic and PCR assays. Seven products intended for daily disinfection in the hospitals were tested against *Clostridium* spp. spore collections. (3) Results: Among 100 isolates, 90 *P. sordellii* were identified, and all strains were devoid of lethal and hemorrhagic toxin genes. Beta-lactam, linezolid, vancomycin, tigecycline, rifampicin, and chloramphenicol all proved effective against isolated strains. Among all strains tested, the spores of *P. sordellii* exhibited remarkable resistance to the tested biocides compared to other *Clostridium* species. The (chlorine-based 0.6%, 30 min), (glutaraldehyde solution 2.5%, 30 min), and (hydrogen peroxide/peracetic acid 3%, 15 min) products achieved the required reduction in spores. (4) Conclusions: Our hospital’s current cleaning and disinfection methods need to be optimized to effectively remove spores from caregivers’ hands, equipment, and surfaces.

## 1. Introduction

Clostridia are composed of a broad spectrum of Gram-positive, spore-forming, and anaerobic bacilli, whose taxonomic classification of genera has been updated. Toxin-producing species can cause mild to life-threatening infections, the most famous being the genus *Clostridium* (*Clostridium botulinum*, *Clostridium perfringens*), the genus *Paeniclostridium* (*Paeniclostridium sordellii*), and the genus *Clostridioides* (*Clostridioides difficile*) [1]. The clostridial spores display intrinsic resistance to high temperatures and biocides and persist for several months on abiotic surfaces [2]. Their environmental stability and antimicrobial tolerance are significant reasons that this group of bacteria can cause severe problems within healthcare settings [3].

*P. sordellii* (previously *Clostridium sordellii*, reclassified in 2016) [4] are ubiquitous in soil, in the gastrointestinal tracts of animals and humans, and in the vaginal microbiota of a modest number of healthy carrier women [5]. As with *C. perfringens*, *P. sordellii* is most often associated with fulminant toxic shock syndrome, sepsis, and gas gangrene in postpartum and post-abortive women, in injection drug use, or after trauma or surgery [6]. *P. sordellii* causes infection in humans sporadically; it is less prevalent than *C. perfringens* infections, but its lethality rate is relatively higher, close to 70% [7]. The infection can develop from endogenous self-contamination or spore transmission from the environment [8]. Generally, this infection is afebrile, and the clinical manifestations include tachycardia, hypotension, leukemoid reaction, hemoconcentration, edema, and hemorrhage [9].

*P. sordellii* shares substantial genomic similarity with *C. difficile* (previously *Clostridium difficile*, reclassified in 2016) [10], but each has specificities regarding the site of infection and clinical manifestation [11]. Pathogenic isolates produce at least five toxins, two of which are essential for virulence, lethal toxin (TcsL) and hemorrhagic toxin (TcsH). TcsH and TcsL are highly similar in their biological activity and their antigenicity to toxin A (TcdA) and toxin B (TcdA) of *C. difficile*, respectively, and they can be revealed through cross-reactivity by *C. difficile* toxin detection [12,13]. These toxins are monoglucosyltransferases, and their main targets are endothelial cells. In addition, numerous studies have reported that non-toxigenic *P. sordellii* strains are associated with invasive infection cases and had cytotoxicity power towards the mammalian cell in vitro [14,15,16,17].

*P. sordellii* infections are severe, with a brief period between the onset of symptoms and death. The only viable treatment option is antibiotic therapy [5]. All *P. sordellii* isolates reported in the literature are highly susceptible to various antibiotics, such as b-lactam, erythromycin, metronidazole, and glycopeptides [5,18]. However, the resistance patterns to clindamycin were different between studies [5].

The presence of spores on surfaces and in the environment in high-risk departments such as intensive care units (ICU) and operating rooms (OR) could be the direct cause of nosocomial infections [19]. The invasive procedures and cutaneous barrier breaking facilitate the penetration and germination of spores in the host and lead to severe complications, such as myonecrosis, gas gangrene, bacteremia, and septicemia [20]. Alkylating agents, oxidizing agents, and chlorine-releasing agents are the most sporicidal disinfectants commonly used in the hospital; despite the success in inactivating spores, routine applications are limited due to their toxic and corrosive properties, and their instability during storage or after preparation influences the quality of disinfection and, more specifically, the elimination of spores [21,22,23].

The goal of the present work was to evaluate the prevalence, and antimicrobial susceptibility, of clostridia strains isolated from environmental surfaces in the University Hospital of Tlemcen (Northwest Algeria). Moreover, molecular investigation of toxin genes was performed on *P. sordellii* isolates, given the large number of isolates obtained in this study.

## 2. Results

### 2.1. Environmental Samples

**Figure 1:**
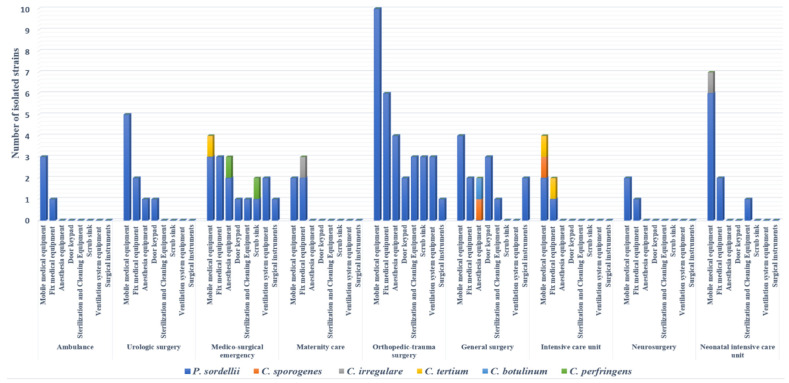
Distribution of clostridia strains isolated in hospital environment according to sampling sites and wards.

One hundred out of 500 (20%) surface samples collected were positive for clostridia strains after culture. Of these 100 isolates, 90 were identified as *P. sordellii*, showing a prevalence of 18%; the other *Clostridium* spp. belonged to seven species, including *Clostridium tertium* (3/100), *C. perfringens* (2/100), *Clostridium irregulare* (2/100), *Clostridium sporogenes* (2/100), and *C. botulinum* (1/100) (Figure 1). Eighty percent of strains were isolated from operating room surfaces, with full concentration in three wards (orthopedic–trauma surgery, surgical emergency, general surgery). Regarding the sampling site, a large number of strains (41%) were isolated from mobile medical equipment (stretchers, drip stands, mayo tables, instrumental tables, work tables, trolleys, mobile radiography systems, mobile ultrasound machines, baby incubators). Ten percent of *P. sordellii* isolates were recovered from cleaning and disinfection equipment and sterile surgical instruments (Figure 1).

### 2.2. Antibiotic Susceptibility Test

Overall, all isolated strains were susceptible to beta-lactam, linezolid, vancomycin, tigecycline, rifampicin, and chloramphenicol (Table 1). In addition, one strain of *C. perfringens* displayed resistance to two antibiotics—metronidazole and clindamycin (MIC 8 and 16 µg/mL, respectively)—and three other strains (two *C. tertium*, one *P. sordellii*) were resistant only to clindamycin (MIC 64 µg/mL).

### 2.3. Testing of Sporicidal Activity

The disinfectant tests were carried out as part of a suspension test under clean conditions, without the addition of organic loads, and in dirty conditions, with the presence of organic loads (3% bovine serum albumin and 0.3% sheep erythrocytes) added to the test solution to mimic the organic contamination in the hospital environment. The effectiveness of the disinfectants was strongly associated with the product composition, the targeted strain, and the conditions of experimentation. Among the seven products tested), three disinfectants—sodium dichloroisocyanurate (D1), glutaraldehyde (D5), and hydrogen peroxide/peracetic acid (D3)—achieved the required reduction in spores for all tested strains under clean conditions (4 log_10_) and under dirty conditions (3 log_10_).

#### 2.3.1. Under Clean Conditions

**Figure 2:**
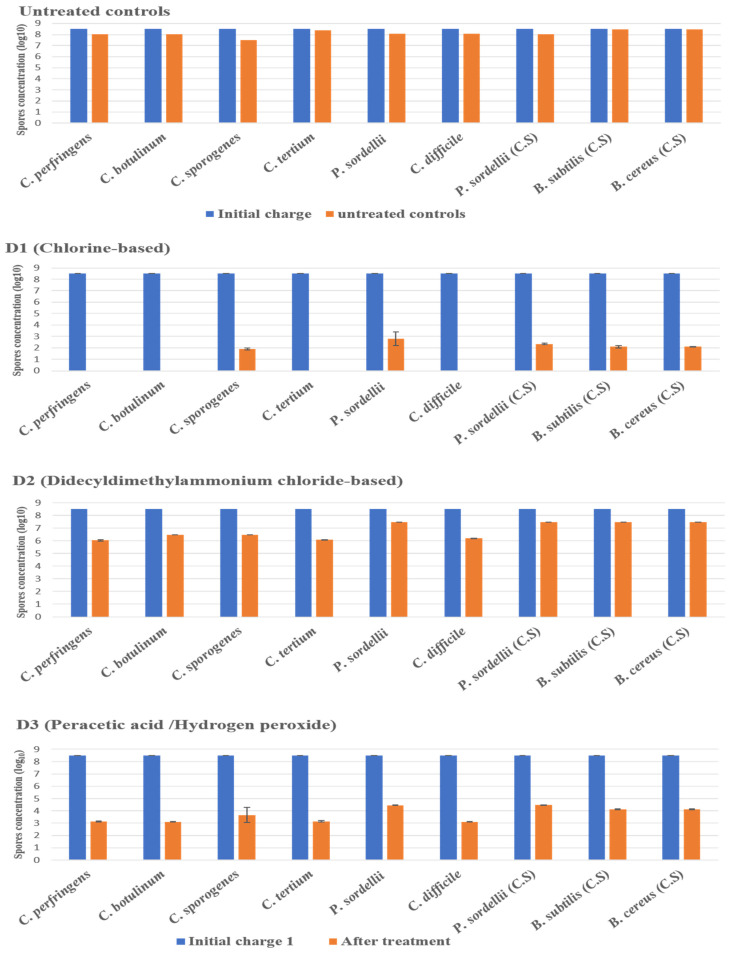
The effect of D1 (chlorine-based), D2 (didecyldimethylammonium chloride-based), and D3 (hydrogen peroxide/peracetic acid) against spores of tested strains using the European and French standard NF EN 14347, in clean conditions with a standard initial bacterial charge of 3.5 × 10^8^ CFU/mL. Untreated sample was used as a control for experimental conditions. The spore suspensions were suspended in sterile deionized water, for 20 min, and then were filtered and incubated. (C.S) indicates reference strains used as control.

**Figure 3:**
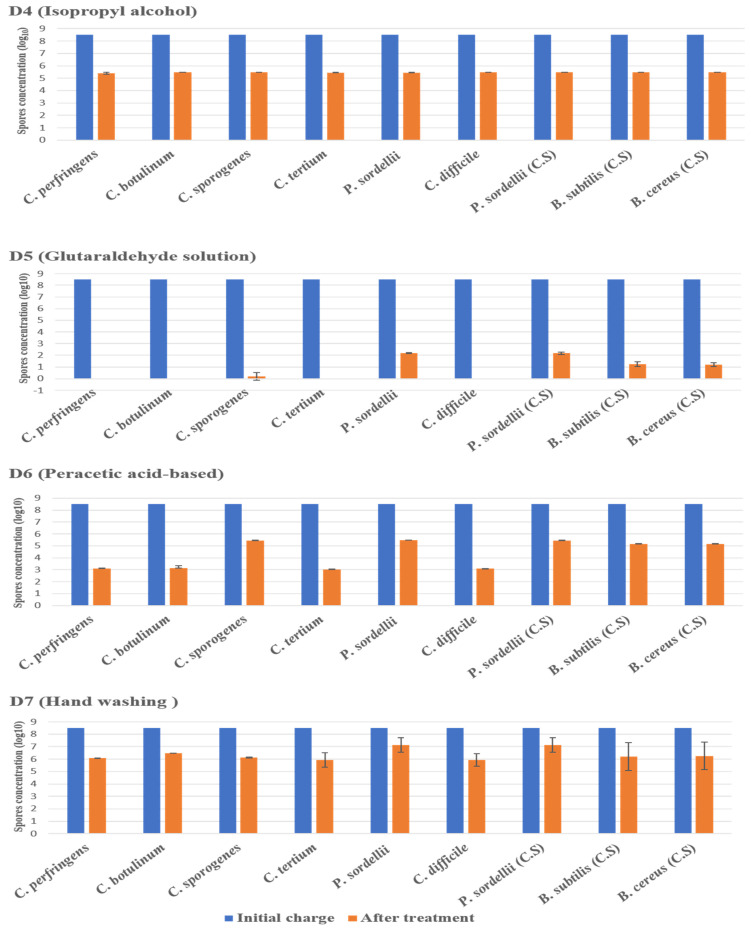
The effect of D4 (isopropyl alcohol), D5 (glutaraldehyde solution), D6 (peracetic acid-based), and D7 (hand washing) against spores of tested strains using the European and French standard NF EN 14347, in clean conditions, with a standard initial bacterial charge of 3.5 × 10^8^ CFU/Ml. (C.S.) indicates reference strains used as control.

The highest log_10_ reduction was measured with D1 (chlorine-based) and D5 (glutaraldehyde solution 2.5%) against spores of all strains tested at a contact time of 30 min. D3 (hydrogen peroxide/peracetic acid) also achieved the recommended reduction rate on all spores. D6 (peracetic acid-based) was found effective against spores of *C. perfringens*, *C. botulinum*, *C. tertium*, and *C. difficile*, giving a reduction of 5.5 log_10_, at the concentration and the contact time recommended by the manufacturer. Remarkably, this disinfectant showed limited sporicidal activity against *P. sordellii*, *C. sporogenes*, and *Bacillus* spp., with a 3 log_10_ reduction (Figure 2 and Figure 3).

The disinfectants D2 (didecyldimethylammonium chloride) and D4 (isopropyl alcohol) and the antiseptic D7 (Figure 2 and Figure 3) were not reliably effective on the spores of all tested strains, showing log_10_ reductions lower than 4.

#### 2.3.2. Under Dirty Conditions

The products D2 and D7 were not included in this experiment because they did not show any activity against all species tested under clean conditions.

**Figure 4:**
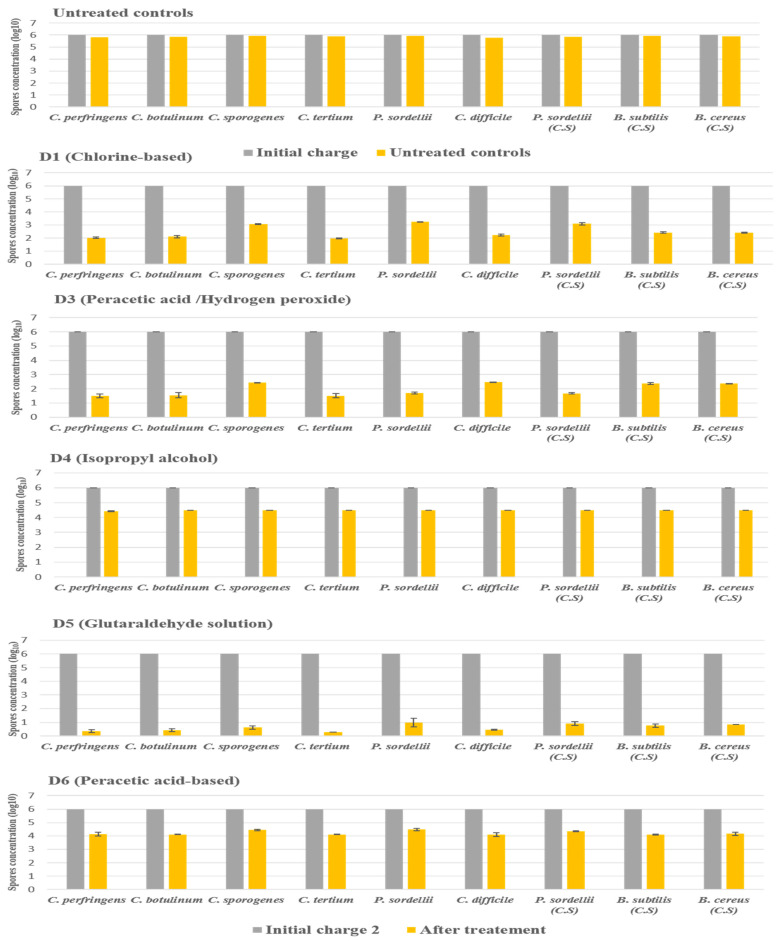
The effect of D1 (chlorine-based), D3 (hydrogen peroxide/peracetic acid), D4 (isopropyl alcohol), D5 (glutaraldehyde solution), and D6 (peracetic acid-based) against spores of tested strains using the European and French standard NF EN 13704, in dirty conditions, with a standard initial bacterial charge of 1 × 10^6^ CFU/mL. Untreated samples were used as a control for experimental conditions. The spore was suspended in sterile deionized water with the addition of (3% bovine serum albumin and 0.3% sheep erythrocytes); after 20 min of contact, the spore suspension was filtered and incubated. (C.S.) indicates reference strains used as control.

In dirty conditions, we noted that the activity of most disinfectants slightly decreased, as illustrated in Figure 4. D5 (glutaraldehyde solution 2.5%) kept its effectiveness against the spores of all strains tested, giving a log_10_ reduction of 5. In contrast, D1 (chlorine-based) lost its activity slightly in the presence of interfering substances, recording a log_10_ reduction of 2.9 against spores of *P. sordellii* and *C. sporogenes*. Indeed, the biocide D3 (hydrogen peroxide/peracetic acid) was always effective on *C. perfringens*, *C. botulinum*, *C. difficile*, and *C. tertium*, giving a log_10_ reduction of 4, with a log_10_ reduction of 3.6 for the four other remaining species.

The D4 disinfectants (isopropyl alcohol) showed a log_10_ reduction of 1.6, and the D6 disinfectants (peracetic acid-based) showed a log_10_ reduction of 1.8. They, therefore, did not achieve the required reductions of 3 log_10_ on all spore species tested after the recommended exposure times under dirty conditions.

### 2.4. Toxin Enzyme Immunoassays (EIA) and PCR Reaction in P. sordellii Isolates

All *P. sordellii* isolates were negative for the TcsL and TcsH toxins, as tested using the C. DIFF QUIK CHEK COMPLETE kit. The results of the designed PCR on the extracted genomic DNA of 288 bacterial strains (Table S2), including 239 clostridia strains, with 26 Gram-negative and 23 Gram-positive strains, were 100% specific and only positive on *P. sordellii* ATCC 9714 and *P. sordellii* VPI 9048 strains harboring either the *tcsL* or *tcsL* and *tcsH* genes. The detection limit of the *tcsL* gene in *P. sordellii* ATCC 9714 and *P. sordellii* VPI 9048 was 37 and 35 CFU/mL, respectively. In addition, the *tcsH* gene’s detection limit in *P. sordellii* VPI 9048 was 40 CFU/mL.

## 3. Discussion

The Environmental Protection Agency (EPA) in the USA has defined spores as the most complicated organism to destroy [24]. In clinical settings, the inanimate environment and healthcare workers’ hands are the most common factors for propagating and transmitting bacterial spores, particularly *C. difficile* spores [25,26]. Moreover, the presence of other clostridial species in a hospital environment that may be more persistent and virulent is also a reality. In this study, numerous *P. sordellii* strains were isolated in surgical wards. However, in high-risk departments, the invasive procedures and cutaneous barrier breaking facilitate the penetration and germination of spores in the host. Several authors revealed that clostridia are commonly implicated in deeper tissue infections, gas gangrenous, myonecrosis, and septicemia [20,27]. *P. sordellii* infections were reported in various pathologies, affecting several anatomical sites, including gynecological infections, peritonitis, endocarditis, pneumonia, arthritis, cellulitis, and myonecrosis [28].

In this study, the contamination of mobile medical equipment such as stretchers, drip stands, mayo tables, instrumental tables, work tables, and trolleys was remarkable; these devices served as potential vectors in spore dissemination in the hospital environment [29]. Ten percent of *P. sordellii* isolates were recovered from cleaning and disinfection equipment and sterile surgical instruments, probably due to intrinsic spore resistance to biocides and high temperatures [30].

*P. sordellii* are known to be susceptible to beta-lactam, metronidazole, and vancomycin [5,31], corresponding to the majority of our isolates. Some strains that showed high MICs to clindamycin were reported [31,32,33]. According to numerous studies, enzyme immunoassays and PCR amplification revealed that all *P. sordellii* isolates were toxin-free [17,34]. The Paloc region of *P. sordellii*, carrying the lethal toxin *tcsL* gene, was found mainly within mobile plasmids (*pCS1* family) [12]. It is worth noting that they are unstable, and the majority of *P. sordellii* isolates can be lost quickly upon collection and subculture, thus compromising their detection. In addition, only sporadic research has succeeded in detecting the *tcsL* gene in a small number of *P. sordellii* strains isolated from different origins: 2/283 strains from rectal and vaginal swabs [35], 1/14 strains from cadaveric tissue donation [36], and 5/44 strains from clinical and veterinary infections [37]. Invasive diseases are also associated with non-toxinogenic strains according to several clinical reports [14,15,16,17].

The present study is among the few studies designed to assess the sporicidal activity of a range of biocides on clostridia spores. We tried to achieve an experimental situation that best simulated the real conditions of disinfection in the hospital by testing under clean and dirty conditions and for times and concentrations of exposure as recommended by the manufacturer and applied by the hygiene teams. Glutaraldehyde solution at 2.5% was the most effective disinfectant on all spore species at a contact time of 30 min, under both clean and dirty conditions. In concordance with many studies, glutaraldehyde is an effective sporicidal agent, used in most hospitals to disinfect thermosensitive and reusable materials [38,39,40]. However, the application of glutaraldehyde solution to surfaces has not been documented, probably because of its relative respiratory and dermal toxicity [41]. A 0.6% sodium dichloroisocyanurate solution in our study effectively inactivated the clostridial spores.

Several authors have stated that chlorine-based products significantly reduce *C. difficile* spores [42,43]. Despite their sporicidal effect, these products can quickly interfere with specific materials and/or organic matter, leading to a loss of their activity and the formation of toxic by-products [44]. In addition, they have a corrosive action on various supports, such as stainless and galvanized steel, and are irritating to manipulators [43,45]. For these reasons, their application is often limited to rooms and surfaces close to patients infected with *C. difficile* [46]. This product is rarely used in the environment of asymptomatic patients, so they are also a potential source of *C. difficile* contamination [47].

Recent publications [48,49,50] report that the peracetic acid/hydrogen peroxide combination reacts synergistically, having robust sporicidal activity, also observed in the presence of an organic load. In our study, this disinfectant achieved a recommended log_10_ reduction on various spores of clostridia species, including virulent strains such as *C. perfringens*, *C. difficile* and *C. botulinum*, *P. sordellii*, and *C. sporogenes*, and spores of aerobic strains. Peracetic acid oxidates the sulfhydryl and sulfur bonds and denatures enzymes and proteins [51]. Many studies have shown that peracetic acid-based disinfectants inactivate bacterial spores of *C. difficile* and *C. sporogenes* in 15 to 30 min [22,52]. The product has proven its effectiveness during our experiment on *C. perfringens*, *C. botulinum*, *C. tertium*, and *C. difficile* spores, although limited sporicidal activity was observed against *P. sordellii* and *C. sporogenes* strains. However, the peracetic acid-based solution decreases their activity in the presence of organic matter and at the time of storage, and it is very corrosive [53,54].

The manufacturer does not report the sporicidal activity of the disinfectants D2 (didecyldimethylammonium chloride) and D4 (isopropyl alcohol). It is already known that alcohol and quaternary ammonium compounds have a narrow spectrum of action and, therefore, a lack of activity against bacterial spores [55,56,57]. We note that disinfectants D2 (didecyldimethylammonium chloride) and D4 (isopropyl alcohol) are extensively used in our hospitals. The widespread use of non-sporicidal disinfectants may encourage bacterial sporulation and participate in their persistence in the environment [25]. In addition, wiping with these products facilitates the spread of bacteria by transferring spores from contaminated sites to clean sites [58]. Healthcare workers’ hands play an essential role in contaminating surfaces and equipment and can transmit bacterial spores between patients.

Many studies have reported that typical hand washing with soap and water does not permanently eliminate *C. difficile* spores [59,60]; our results confirmed these findings. Recent research suggests new approaches that may be more effective against clostridia spores, such as soaking or wiping hands with electrochemically activated saline solution, generating hypochlorous acid (Vashe) [61], and washing hands with sand- or oil-based products [62].

## 4. Materials and Methods

### 4.1. Samples Collection

Over two years (2016 to 2018), a total of 500 surface sample swabs were collected from nine departments of the University Hospital of Tlemcen (urologic surgery, surgical emergency, gynecologic surgery, orthopedic–trauma surgery, general surgery, neurosurgery, intensive care unit, neonatal intensive care unit, ambulance).

A variety of areas in these high-risk units were swabbed after routine cleaning, including mobile equipment (stretchers, drip stands, mayo tables, instrumental tables, work tables, trolleys, mobile radiography systems, ultrasound machines, baby incubators), fix equipment (passbox, operating tables, storage racks, surgical lighting, computers), anesthesia equipment, door keypads, sterilization and cleaning equipment, scrub sinks, ventilation system equipment, and surgical instruments.

### 4.2. Culture and Bacterial Identification

The swabs were suspended and incubated at 37 °C for 48 h in BHI (Laboratoire Conda S.A, Torrejón de Ardoz, Spain). Then, the enrichment broth was heated for 10 min at 80 °C to destroy the vegetative cells and activate the spore formation. Then, 100 µL was cultured on Brazier’s agar without added selective agents (D-cycloserine and cefoxitin) and on 7% sheep blood agar, incubated at 37 °C for 48 h/72 h in an anaerobic atmosphere using the GENbag anaer (bioMérieux, Marcy l’Etoile, France). The bacterial identification was performed using matrix-assisted laser desorption/ionization time-of-flight (MALDI-TOF) mass spectrometry (MS) (Microflex™; Bruker Daltonic, Bremen, Germany) as previously described [63] and confirmed by 16S rRNA sequencing.

### 4.3. Antibacterial Susceptibility Testing

Antimicrobial drug susceptibility was determined using the E-test method. Minimum inhibitory concentration (MIC) results were interpreted using breakpoints recommended by the Antibiogram Committee of the French Microbiology Society and European Committee on Antimicrobial Susceptibility Testing (CA-SFM/EUCAST 2019) https://www.sfm-microbiologie.org/2019/01/07/casfm-eucast-2019/ (accessed on 11 April 2020). The following antibiotics were selected for susceptibility testing: amoxicillin (AC), amoxicillin–clavulanic acid (XL), piperacillin–tazobactam (T/P), imipenem (IP), ertapenem (ETP), clindamycin (CM), metronidazole (MZ), linezolid (LZ), vancomycin (VA), tigecycline (TGC), rifampicin (RI), chloramphenicol (CL) (Ia2, Montpellier, France).

### 4.4. Spore Preparation and Chosen Biocides

We evaluated the efficacity of biocides for the inactivation of clostridial spores on a collection of nine bacterial strains: seven clostridia species strains and two *Bacillus* spp. strains. Additional information regarding all strains tested is presented in Table 2. Clostridia spores were prepared as described by Perez et al. (2011) [64] with some modifications. *Bacillus* spp. spores were prepared according to the ASTM E2197-11 standard [43]. The spores’ concentration was measured by traditional hemacytometer counting. The rate of germinating spores was estimated by counting the number of germinated spores after culture versus the total count of spores.

Seven disinfectant products routinely used in Algerian and French hospital settings were evaluated, including six disinfectants used for the disinfection of surfaces, thermosensitive equipment, instrumentation, and medical devices, and one product used for hand washing among medical staff. Active ingredients and commercial forms are reported in Table 3. The contact times and concentrations were applied according to the manufacturer’s instructions for sporicidal products and as used by the hygiene teams for non-sporicidal products (Table 3). The D2 and D7 products were supplied in ready-to-use liquid form and tested only under clean conditions. The other products were provided in liquid and powder formulations and were prepared by mixing with sterile deionized water.

### 4.5. Testing of Sporicidal Activity

The disinfectant tests were carried out as part of a suspension test following the guidelines of the European and French standard NF EN 14347 under clean conditions, without the addition of organic loads, and according to the European and French standard NF EN 13704, in dirty conditions, with the presence of organic loads (3% bovine serum albumin and 0.3% sheep erythrocytes) added to the test solution to mimic organic contamination in the hospital environment [55].

The sporicidal activity was determined in triplicate. For each experiment, a standard initial bacterial charge of 3.5 × 10^8^ CFU/mL and 1 × 10^6^ CFU/mL was used for clean and dirty conditions, respectively, according to the European and French standards mentioned above. After the contact time (Table 3), the suspensions were filtered using membrane filtration (0.22 µm) and washed three times with phosphate-buffered saline (PBS) to eliminate any effects of residual antimicrobial components.

After 48 h of incubation, the number of viable spores was determined using the standard colony count method. Log_10_ reductions were calculated by comparing log_10_ CFU recovered from disinfectant solutions to untreated controls (spores suspended in deionized water). The sporicidal activity was defined as a reduction of 4 log_10_ under clean conditions and 3 log_10_ under dirty conditions.

### 4.6. Enzyme Immunoassays and PCR for the Detection of P. sordellii Toxins

Enzymatic research on *P. sordellii* toxins was performed with the C. DIFF QUIK CHEK COMPLETE assay kit (TECHLAB, Blacksburg, VA, USA). The only non-*C. difficile* micro-organisms detected by the C. DIFF QUIK CHEK COMPLETE^®^ test were *P. sordellii* strains that produce toxins TcsL and TcsH, which are homologous to toxins TcdA and TcdB, from *C. difficile*, respectively [65]. Isolated colonies were suspended in the dilution buffer and tested according to the manufacturer’s protocol; *P. sordellii* VPI 9048 strain was used as a positive control.

For the molecular detection of *P. sordellii* lethal toxin (TcsL) and hemorrhagic toxin (TcsH), two sets of primers were designed to amplify an internal fragment of 1221 bp size and 1430 bp size, respectively (Table S1). The primers were validated and optimized using DNA extracted from 288 bacterial strains, including 239 clostridia strains, 26 Gram-negative and 23 Gram-positive strains, and two control strains—*P. sordellii* ATCC 9714 and *P. sordellii* VPI 9048 (Table S2).

## 5. Conclusions

*P. sordellii* is capable of causing severe and often fatal infections. Our study is the first to report a high prevalence of *P. sordellii* isolates in the hospital environment in Algeria. This situation is probably due to a deficiency in the cleaning of surfaces and instruments, leading to the massive presence of spores in our hospital. This situation needs to be controlled quickly and significantly, since these strains may rapidly acquire the genes of toxins and antibiotic resistance. We observed that the spores of *P. sordellii* showed higher resistance to all products tested compared to spores of other clostridia species and *Bacillus* spp. Our results reveal that sodium dichloroisocyanurate (0.6%), glutaraldehyde (2.5%), and hydrogen peroxide/peracetic acid (3%) were the most effective and should be prioritized for routine disinfection in the hospital. Our hospital’s current cleaning and disinfection methods need to be optimized to effectively remove spores from caregivers’ hands, equipment, and surfaces.

## Figures and Tables

**Table 1:** Minimum inhibitory concentration ranges and interpretations of the tested antibiotics to isolated strains from environmental samples.

Species	N° of Isolates	AC	XL	TP	IP	ETP	CM	MZ	LZ	VA	TGC	RI	CL
MIC range (µg/mL)	100	0.125–0.25	0.047–0.25	0.047–0.19	0.008–0.012	0.008–0.012	0.25–64	0.032–8	0.064–0.25	0.032–0.094	0.047–0.125	0.032–0.094	0.006–0.016
*P. sordellii*	89	S	S	S	S	S	S	S	S	S	S	S	S
*P. sordellii*	1	S	S	S	S	S	R	S	S	S	S	S	S
*C. tertium*	2	S	S	S	S	S	R	S	S	S	S	S	S
*C. tertium*	1	S	S	S	S	S	S	S	S	S	S	S	S
*C. perfringens*	1	S	S	S	S	S	S	S	S	S	S	S	S
*C. perfringens*	1	S	S	S	S	S	R	R	S	S	S	S	S
*C. irregulare*	2	S	S	S	S	S	S	S	S	S	S	S	S
*C. sporogenes*	2	S	S	S	S	S	S	S	S	S	S	S	S
*C. botulinum*	1	S	S	S	S	S	S	S	S	S	S	S	S

**Abbreviations:** Amoxicillin (AC), Amoxicillin–Clavulanic Acid (XL), Piperacillin–Tazobactam (T/P), Imipenem (IP), Ertapenem (ETP), Clindamycin (CM), Metronidazole (MZ), Linezolid (LZ), Vancomycin (VA), Tigecycline (TGC), Rifampicin (RI), Chloramphenicol (CL), Resistant (R), Sensitive (S).

**Table 2:** List of strains tested and their origins, with concentration and percentage of germination of spores in stock solutions.

Tested Strains	Origins	Spore Concentration (mL^−1^)	Spore Germination Percentage (%)
*C. perfringens*	Hospital environment, surface (UHT)	1.24 × 10^8^	86.29%
*C. botulinum*	Hospital environment, surface (UHT)	1.14 × 10^8^	90.35%
*C. sporogenes*	Hospital environment, surface (UHT)	3.20 × 10^7^	93.75%
*C. tertium*	Hospital environment, surface (UHT)	2.67 × 10^8^	93.26%
*P. sordellii*	Hospital environment, surface (UHT)	1.24 × 10^8^	91.13%
*C. difficile*	CSUR (P8093)	1.32 × 10^8^	90.15%
*P. sordellii*	DSM 2141	1.19 × 10^8^	92.44%
*B. subtilis*	DSM 347	3.17 × 10^8^	94.63%
*B. cereus*	DSM 106266	2.97 × 10^8^	93.60%

**Table 3:** Product forms, ingredients, use concentrations, and contact times.

Product	Disinfectant Forms	Active Ingredient	Concentration	Contact Time and Type of Application	Spectrum of Activity
D1	Effervescent tablet	Sodium dichloroisocyanurate (81%), (N° CAS 2893-78-9), pH: 7.4	0.6%	30 min Surface Disinfection	Fungicidal, virucidal, sporicidal, and bactericidal
D2	Spray	Didecyldimethylammonium chloride 3 mg/g (N° CAS 7173-51-5), pH: 6 ± 0.5	Ready to use	30 min Surface and medical device disinfection	Fungicidal, virucidal, and bactericidal
D3	Liquid concentrated solution	Hydrogen peroxide 255.9 mg/g (N° CAS 7722-84-1), Peracetic acid 48 mg/g (N° CAS 79-21-0), pH: 4	3%	15 min Surface and instrument disinfection	Fungicidal, virucidal, sporicidal, and bactericidal
D4	Liquid concentrated solution	Isopropyl alcohol (N° CAS 67-63-0), pH: 7.4	90%	20 min Instrument disinfection	Virucidal and bactericidal
D5	Liquid concentrated solution	Glutaraldehyde (N° CAS 111-30-8), pH: 5.2	2.5%	30 min at 20 °C Instrument disinfection	Fungicidal, virucidal, sporicidal, and bactericidal
D6	Granules	Peracetic acid 750 ppm (N° CAS 79-21-0), Dimethylammonium chloride 0.012% (N° CAS: 85409-22-9), pH: 9.3	0.5%	15 min Cleaning and disinfection of floors and surfaces	Fungicidal, virucidal, sporicidal, and bactericidal
D7	Liquid	Lauramphocarboxyglycinate, Sodium lauryl sulfate, Linoleamide DEA, benzyl alcohol, sodium benzoate, pH: 6.5	Ready to use	30 s Hand washing	No

## Data Availability

Not applicable.

## Supplementary Materials

The following supporting information can be downloaded at: https://www.mdpi.com/article/10.3390/antibiotics11010038/s1, Supplementary Table S1: List of primers designed and validated in this study. Supplementary Table S2: List of the bacterial strains tested for the specificity of the designed PCR assay.
