# Supplementary material for: Prevalence and Antimicrobial Resistance of Paeniclostridium sordellii in Hospital Settings
Source: Antibiotics (Basel). 2021 Dec 29;11(1):38. doi: 10.3390/antibiotics11010038 (PMC8772839; doi:10.3390/antibiotics11010038)
Supplement: Supplementary file 1 [file antibiotics-11-00038-s001.zip › Table S1.pdf]

**Table S1.** List of primers designed and validated in this study.

| . | Primer and Probe Name | Primer and Probes Sequence | Length (bp) | Amplicon Size (bp) | Reference |
|---|---|---|---|---|---|
| **Standard PCR** | *tcsL-F* | TGCTCCTGCTGGTACACTTG | 20 | 1221 | This study |
| | *tcsL -R* | TCCCCCTCAAAATTCTCATC | 20 | | |
| | *tcsH -F* | TGGAGTTTTGATCAAGCAAGCG | 22 | 1430 | This study |
| | *tcsH -R* | AGTTGGGCATACAGTTGAACCT | 22 | | |
