# Supplementary material for: Prevalence and Antimicrobial Resistance of Paeniclostridium sordellii in Hospital Settings
Source: Antibiotics (Basel). 2021 Dec 29;11(1):38. doi: 10.3390/antibiotics11010038 (PMC8772839; doi:10.3390/antibiotics11010038)
Supplement: Supplementary file 1 [file antibiotics-11-00038-s001.zip › Table S2.pdf]

Table S2. List of the bacterial strains tested for the specificity of the designed PCR assay.

Type of Bacteria	Bacterial Species	Number of Strains	Origin	PCR	
				tcsL	*tcsH*
**Gram-positive bacteria**	*Staphylococcus aureus*	1	Marseille	-	-
	Staphylococcus epidermidis	1	Marseille	-	-
	Streptococcus agalactiae	1	Marseille	-	-
	Streptococcus pneumoniae	1	Marseille	-	-
	Staphylococcus haemolyticus	1	Marseille	-	-
	Staphylococcus capitis	1	Marseille	-	-
	Staphylococcus lugdunensis	1	Marseille	-	-
	Streptococcus mitis	1	Marseille	-	-
	Staphylococcus warneri	1	Marseille	-	-
	Corynebacterium striatum	1	Marseille	-	-
	Staphylococcus saprophyticus	1	Marseille	-	-
	Corynebacterium jeikeium	1	Marseille	-	-
	Staphylococcus simulans	1	Marseille	-	-
	Staphylococcus pasteuri	1	Marseille	-	-
	Corynebacterium amycolatum	1	Marseille	-	-
	Bacillus cereus	1	Marseille	-	-
	Staphylococcus cohnii	1	Marseille	-	-
	Streptococcus salivarius	1	Marseille	-	-
	Streptococcus equinus	1	Marseille	-	-
	Corynebacterium propinquum	1	Marseille	-	-
	Micrococcus luteus	1	Marseille	-	-
	Streptococcus dysgalactiae	1	Marseille	-	-
	Staphylococcus hominis	1	Marseille	-	-
**Total of strains = 23**					
**Gram-negative bacteria**	*Proteus mirabilis*	1	Marseille	-	-
	Citrobacter freundii	1	Marseille	-	-
	Achromobacter xylosoxidans	1	Marseille	-	-
	Enterobacter cloacae	1	Marseille	-	-
	Bacteroides fragilis	1	Marseille	-	-
	Moraxella catarrhalis	1	Marseille	-	-
	Proteus vulgaris	1	Marseille	-	-
	Providencia stuartii	1	Marseille	-	-
	Haemophilus parainfluenzae	1	Marseille	-	-
	Klebsiella pneumonia	1	Marseille	-	-
	Pseudomonas aeruginosa	1	Marseille	-	-
	Enterobacter kobei	1	Marseille	-	-
	Enterobacter asburiae	1	Marseille	-	-
	Hafnia alvei	1	Marseille	-	-
	Raoultella ornithinolytica	1	Marseille	-	-
	Citrobacter braakii	1	Marseille	-	-
	Escherichia coli	1	Marseille	-	-
	Pasteurella multocida	1	Marseille	-	-
	Stenotrophomonas maltophilia	1	Marseille	-	-
	Morganella morganii	1	Marseille	-	-
	Citrobacter koseri	1	Marseille	-	-
	Enterobacter aerogenes	1	Marseille	-	-
	Haemophilus influenzae	1	Marseille	-	-

	Klebsiella oxytoca	1	Marseille	-	-
	Acinetobacter baumannii	2	Marseille	-	-
**Total of strains = 26**					
***Clostridium* spp. from CSUR collection**	*Clostridium aerotolerans*	1	CSUR	-	-
	Clostridium aldenense	1	CSUR	-	-
	Clostridium baratii	1	CSUR	-	-
	Clostridium bifermentans	1	CSUR	-	-
	Clostridium butyricum	4	CSUR	-	-
	Clostridium cadaveris	1	CSUR	-	-
	Clostridium clostridioforme	1	CSUR	-	-
	Clostridium difficile (TcdA)	12	CSUR	-	-
	Clostridium difficile (TcdB)	10	CSUR	-	-
	Clostridium difficile (TcdA +TcdB)	30	CSUR	-	-
	Clostridium difficile	45	CSUR	-	-
	Clostridium disporicum	1	CSUR	-	-
	Clostridium fallax	1	CSUR	-	-
	Clostridium ghonii	1	CSUR	-	-
	Clostridium histolyticum	1	CSUR	-	-
	Clostridium innocuum	1	CSUR	-	-
	Clostridium neonatale	1	CSUR	-	-
	Clostridium paraputrificum	1	CSUR	-	-
	Clostridium perfringens	4	CSUR	-	-
	Clostridium ramosum	1	CSUR	-	-
	Clostridium saudii	1	CSUR	-	-
	Clostridium septicum	1	CSUR	-	-
	Clostridium sordellii	7	CSUR	-	-
	Clostridium sphenoides	1	CSUR	-	-
	Clostridium sporogenes	1	CSUR	-	-
	Clostridium subterminale	1	CSUR	-	-
	Clostridium symbosium	1	CSUR	-	-
	Clostridium tertium	4	CSUR	-	-
	Clostridium tetani	1	CSUR	-	-
***Clostridium* spp. from this study**	*P. sordellii*	90	Tlemcen	-	-
	Clostridium tertium	3	Tlemcen	-	-
	Clostridium perfringens	2	Tlemcen	-	-
	Clostridium irregulare	2	Tlemcen	-	-
	Clostridium sporogenes	2	Tlemcen	-	-
	Clostridium botulinum	1	Tlemcen	-	-
**Total of strains = 237**					
**Reference strains**	*Paeniclostridium sordellii (TcsL + TcsH)*	1	VPI 9048	+	+
	Paeniclostridium sordellii (TcsL)	1	ATCC 9714	+	-
**Total of strains = 2**					

CSUR : Collection de Souches de l'Unité des Rickettsies. ATCC: American Type Culture Collection.
